# Neurocognitive Decline in Rapid Eye Movement (REM)-Predominant Obstructive Sleep Apnea (OSA) Versus Non-rapid Eye Movement (NREM)-Predominant OSA: A Comparative Study

**DOI:** 10.7759/cureus.93158

**Published:** 2025-09-24

**Authors:** Sudeeksha Purushotham, Archana Baburao, Parinita Suresh, Ponnathota Vindhya

**Affiliations:** 1 Department of Respiratory Medicine, RajaRajeswari Medical College and Hospital, Bangalore, IND

**Keywords:** diagnosis, neurocognitive decline, nrem-osa, rem-osa, sleep apnea

## Abstract

Objective: The present study aims to investigate the distribution and characteristics of obstructive events across rapid eye movement (REM) and non-rapid eye movement (NREM) sleep stages and to assess their clinical and neurocognitive implications in patients with obstructive sleep apnea (OSA) in an Indian population.

Methodology: This comparative study was conducted at the Department of Respiratory Medicine, Rajarajeswari Medical College and Hospital, Bangalore, from January to June 2025. It included 50 suspected OSA patients, screened using the STOP-BANG questionnaire. All participants underwent level 1 polysomnography and were classified as having either REM-predominant or NREM-predominant OSA based on the American Academy of Sleep Medicine (AASM) criteria. Neurocognitive and depression assessments were performed using the Mini-Cog test and the Patient Health Questionnaire-9 (PHQ-9). Statistical analysis was conducted using IBM SPSS Statistics for Windows, Version 22.0. The Mann-Whitney U test was applied for group comparisons, and a p-value of <0.05 was considered statistically significant.

Results: The study included 50 patients, comprising 30 (60%) male patients and 20 (40%) female patients. Among the male patients, 27 (90%) had NREM-predominant OSA, while three (10%) had REM-predominant OSA. Among the female patients, 13 (65%) had NREM-predominant OSA, whereas seven (35%) had REM-predominant OSA. Female patients were more frequently presented with fatigue, whereas snoring was more commonly reported among male patients. The mean age was 50.63 ± 16.19 years for the NREM group and 52.80 ± 13.02 years for the REM group. The mean body mass index (BMI) was 30.47 ± 5.49 kg/m² in NREM OSA and 53.27 ± 63.43 kg/m² in REM OSA. The mean heart rate was 72.05 ± 8.22 beats per minute (bpm) in NREM OSA and 75.70 bpm in REM OSA. The NREM apnea-hypopnea index (AHI) was 5.27 ± 2.63 in REM OSA and 35.56 ± 29.17 in NREM OSA, with significantly higher values in the latter (p = 0.0001). The Mini-Cog score was considerably lower in REM OSA (1.30 ± 1.34) compared to NREM OSA (4.00 ± 1.28) (p = 0.0001). The PHQ-9 score was significantly higher in REM OSA (12.40 ± 4.20) than in NREM OSA (3.48 ± 2.70) (p = 0.0001).

Conclusion: The detrimental effects of REM-predominant OSA on cognitive function are linked to reduced intracranial oxygen supply, fragmented sleep, and elevated oxidative stress and inflammation. Maintaining continuous positive airway pressure use in the early morning hours, when patients are most prone to discontinuation, is essential for optimal management. Our study emphasizes the need for neurocognitive screening in all newly diagnosed REM-predominant OSA patients to help prevent serious comorbidities. Early and appropriate intervention may mitigate the progression of neurocognitive impairment.

## Introduction

Obstructive sleep apnea (OSA) syndrome is a common sleep-related breathing disorder characterized by recurrent episodes of complete (apnea) or partial (hypopnea) upper airway obstruction during sleep, leading to intermittent hypoxemia and sleep fragmentation due to frequent arousals [1]. These obstructive events result from repetitive partial or complete collapse of the upper airway. The severity of OSA is typically quantified using the apnea-hypopnea index (AHI), defined as the number of apneas and hypopneas per hour of sleep [1].

While upper airway collapse can occur during rapid eye movement (REM) and non-rapid eye movement (NREM) sleep, neurophysiological mechanisms increase vulnerability during REM sleep. During REM sleep, excitatory noradrenergic and serotonergic signals to the upper airway motor neurons are withdrawn. This reduces the tone of the pharyngeal dilator muscles, increasing the likelihood and severity of airway collapse [2]. Consequently, patients with OSA may experience more frequent, prolonged, and severe obstructive events with greater oxygen desaturation during REM sleep [3].

In some individuals, respiratory disturbances occur predominantly during REM sleep. A commonly used diagnostic criterion for REM-predominant OSA is an AHI-REM to AHI-NREM ratio greater than 2 [4]. Reported prevalence ranges from 10% to 36%, varying with study population characteristics and operational definitions [1-3]. This phenotype is more frequently observed in female sex, younger adults, children, and individuals with mild to moderate OSA [2,4].

REM-predominant OSA is increasingly recognized as a distinct clinical phenotype, often associated with younger age, female sex, and higher body mass index (BMI) [5]. The high susceptibility to airway obstruction during REM sleep is attributed to reduced upper airway muscle tone compared to NREM sleep [5]. However, OSA may also occur predominantly during NREM sleep or be evenly distributed across stages [5,6]. The pathophysiology and prevalence of REM-versus NREM-predominant OSA remain incompletely understood [6]. Ventilatory instability during NREM sleep transitions may contribute to irregular respiratory patterns and trigger obstructive events [7], while mechanistic studies of respiratory control during REM sleep remain limited [8]. These gaps highlight the importance of detailed polysomnographic assessment to differentiate REM and NREM-predominant OSA phenotypes.

Adequate and good-quality sleep is essential for maintaining neurocognitive function [9]. OSA disrupts this process through recurrent hypoxia, hypercapnia, and sleep fragmentation, which have been linked to deficits in attention, memory, executive function, language, psychomotor speed, and visuospatial abilities [9,10]. Recurrent arousals contribute to cognitive decline by causing chronic sleep deprivation [10]. Deep NREM (slow-wave) sleep is critical for memory consolidation and may modulate neurodegenerative processes by influencing pathological proteins such as beta-amyloid, tau, and alpha-synuclein [11]. Experimental studies have shown that sleep deprivation increases, and sleep promotion decreases, the levels of these neurotoxic proteins in brain tissue, interstitial fluid, and cerebrospinal fluid [12]. REM sleep deprivation has also been linked to poorer cognitive outcomes, suggesting that disturbances in this stage may negatively affect daytime functioning [13].

Despite growing evidence linking OSA to cognitive impairment, research specifically focused on the cognitive consequences of REM-predominant OSA is limited. In this context, the present study aims to examine the distribution and characteristics of obstructive events across REM and NREM sleep stages and assess their clinical and neurocognitive implications in an Indian patient population.

## Materials and methods

This comparative study was conducted from January to June 2025 in the Department of Respiratory Medicine, Rajarajeswari Medical College and Hospital, Bangalore, affiliated with Dr. M.G.R. Educational and Research Institute. A total of 50 patients who met the inclusion criteria were enrolled. Patients were recruited as consecutive referrals from the sleep clinic during the study period to ensure reproducibility and minimize selection bias. All participants were screened for symptoms of obstructive sleep apnea using the STOP-Bang questionnaire [14]. A cut-off score of ≥3 on the STOP-Bang was applied to define high-risk individuals eligible for polysomnography. The STOP-Bang questionnaire is free for clinical and research use under the Creative Commons Attribution-NonCommercial-NoDerivatives 3.0 (CC BY-NC-ND 3.0) license [14]. Permission for its use in this study was formally requested from Dr. Frances Chung, the original developer, on August 12, 2025. Written informed consent was obtained from all participants, and ethical approval was granted by the Institutional Ethics Committee (RRMCH-IEC/15/2025). The sample size of 50 was determined pragmatically based on the expected number of eligible referrals during the six-month recruitment period, and it is in line with a similar study of Yusop et al. [15]. Although a formal power calculation was not feasible due to limited prior data on REM-predominant OSA and cognition, this number was considered adequate for preliminary comparative analysis.

All participants underwent level 1 polysomnography. Parameters recorded included electroencephalogram (EEG), electrooculogram (EOG), electromyogram (EMG), electrocardiogram (ECG), nasal and oral airflow, thoracic and abdominal respiratory movements, and peripheral capillary oxygen saturation (SpO₂). Apneas and hypopneas were identified and scored according to the 2014 edition of the American Academy of Sleep Medicine (AASM) scoring manual [16]. Scoring was performed manually by a board-certified sleep physician with over five years of clinical experience in sleep medicine. A second certified physician independently reviewed 20% of the studies to further enhance reliability, with inter-rater agreement assessed (κ = 0.87). Patients were classified as having rapid eye movement (REM)-predominant OSA if the REM sleep duration was ≥30 minutes, the ratio of AHI during REM to AHI during NREM sleep was >2, and the NREM AHI was <15 events per hour.

Patients were excluded if they had REM sleep duration <30 minutes, declined to complete the questionnaires, or had pre-existing neurodegenerative disorders (e.g., Alzheimer’s disease, Parkinson’s disease), pre-existing depression, or mental disabilities that could compromise the reliability of assessments. Additional exclusion criteria included the use of medications known to impair cognition (e.g., opioids, antidepressants, benzodiazepines, antihistamines), substance abuse, or a diagnosis of obesity hypoventilation syndrome (OHS).

Following polysomnography, patients were categorized into two groups: REM-predominant OSA and NREM-predominant OSA. The Mini-Cog test was administered in the morning following polysomnography, under standardized quiet room conditions, to minimize the effects of diurnal variation and fatigue. Cognitive function was assessed using the Mini-Cog test [17], which comprises three components: three-word registration, a clock-drawing task, and three-word recall. Scores range from 0 to 5, with lower scores indicating greater neurocognitive impairment. The Mini-Cog is free to use and reproduce for clinical and research purposes without permission, as long as an appropriate citation is given (Copyright © 2000 John Wiley & Sons, Ltd.) [17].

Depression severity was evaluated using the Patient Health Questionnaire-9 (PHQ-9) [18]. Participants scoring 1-4 were classified as having minimal depression, 5-9 mild depression, 10-14 moderate depression, 15-19 moderately severe depression, and 20-27 severe depression. This scale facilitates the quantification of symptom severity and supports clinical decision-making. As per the official statement, the PHQ-9, developed by Drs. Robert L. Spitzer, Janet B.W. Williams, Kurt Kroenke, and colleagues with an educational grant from Pfizer Inc., may be reproduced, translated, displayed, or distributed for clinical or research purposes without permission [18]. Fatigue was evaluated using the Fatigue Severity Scale (FSS) [19], a validated nine-item questionnaire scored from 1 to 7, with higher scores indicating greater fatigue severity. Fatigue assessment was conducted in the morning after polysomnography under standardized conditions to reduce variability and allow exploration of potential sex-related differences. Body mass index (BMI) was calculated as weight (kg) divided by height squared (m²), and extreme values were cross-checked for data entry errors.

Statistical analysis

Data were analyzed using Microsoft Excel and IBM SPSS Statistics for Windows, Version 22 (Released 2013; IBM Corp., Armonk, New York, United States). Results are expressed as mean ± standard deviation (SD). The chi-square test was used to assess differences between groups. A p-value <0.05 was considered statistically significant.

## Results

Our study included 50 patients, comprising 30 (60%) male patients and 20 (40%) female patients. Among the male patients, 27 (90%) had NREM-predominant OSA, while three (10%) had REM-predominant OSA. Among the female patients, 13 (65%) had NREM-predominant OSA, whereas seven (35%) had REM-predominant OSA (Table 1).

**Table 1:** Gender distribution among patients with rapid eye movement-predominant versus non-rapid eye movement-predominant obstructive sleep apnea NREM: non-rapid eye movement; REM: rapid eye movement; OSA: obstructive sleep apnea * shows a significant p-value; p<0.05 was considered significant

Parameters	NREM OSA N (%)	REM OSA N (%)	Total N (%)	Chi-square	p-value
Sex				4.688	0.030*
Male	27 (67.5%)	3 (30%)	30 (60%)		
Female	13 (32.5%)	7 (70%)	20 (40%)		
Total	40 (100%)	10 (100%)	50 (100%)		

As shown in Table 2, female patients most commonly presented with fatigue, whereas male patients more frequently reported snoring.

**Table 2:** Symptoms in male vs female OSA patients REM-OSA: rapid eye movement obstructive sleep apnea; NREM: non-rapid eye movement obstructive sleep apnea

	Predominant symptoms	Predominant type of OSA
Female	Fatigue	REM-OSA
Male	Snoring	NREM -OSA

Table 3 indicates that the mean age was 50.63 ± 16.19 years for the NREM OSA group and 52.80 ± 13.02 years for the REM OSA group. The mean BMI was 30.47 ± 5.49 kg/m² in the NREM OSA group and 32.5 ± 6.4 kg/m² in the REM OSA group. The mean heart rate was 72.05 ± 8.22 bpm in the NREM OSA group and 75.70 ± 7.18 bpm in the REM OSA group.

**Table 3:** Comparison of age, BMI and heart rate in REM-OSA vs NREM-OSA patients BMI: body mass index; Bpm: beats per min; EM-OSA: rapid eye movement obstructive sleep apnea; NREM-OSA: non-rapid eye movement obstructive sleep apnea

Parameters	NREM-OSA (Mean ± SD)	REM-OSA (Mean ± SD)	t-value	p-value
Age (years)	50.63 ± 16.19	52.80 ± 13.02	0.393	0.696
BMI (kg/m²)	30.47 ± 5.49	32.5 ± 6.4	1.136	0.285
Heart rate (bpm)	72.05 ± 8.93	75.70 ± 7.18	1.197	0.237

Table 4 compares key sleep parameters between REM-OSA and NREM-OSA groups. Wake after sleep onset was 119.06 ± 100.03 minutes in REM-OSA versus 79.53 ± 60.29 minutes in NREM-OSA (p = 0.149). REM latency averaged 126.30 ± 113.62 minutes in REM-OSA and 152.01 ± 198.28 minutes in NREM-OSA (p = 0.762). The NREM-AHI was significantly higher in NREM-OSA (35.56 ± 29.17 events/hour) than REM-OSA (5.27 ± 2.63 events/hour, p = 0.0001). Conversely, the REM-AHI/NREM-AHI ratio was significantly elevated in REM-OSA (4.89 ± 2.89) versus NREM-OSA (1.76 ± 2.13, p = 0.0001). Overall, AHI was significantly lower in REM-OSA (7.65 ± 3.09 events/hour) than in NREM-OSA (38.61 ± 28.77 events/hour, p = 0.0001). Mean oxygen saturation during NREM and REM sleep and arousal indices did not differ significantly between groups. The percentage of total sleep time with SpO₂ < 90% during REM was higher in REM-OSA (24.11 ± 27.11%) than NREM-OSA (12.01 ± 13.31%), but this difference was not statistically significant (p = 0.083).

**Table 4:** Sleep study parameters in REM OSA vs NREM OSA REM: rapid eye movement; NREM: non-rapid eye movement; OSA: obstructive sleep apnea; AHI: apnea–hypopnea index; REM-AHI: apnea–hypopnea index during rapid eye movement sleep; NREM-AHI: apnea–hypopnea index during non-rapid eye movement sleep; REM-AHI/NREM-AHI: ratio of apnea–hypopnea index in REM sleep to that in NREM sleep; Overall AHI: apnea–hypopnea index averaged over total sleep time; SPO2: peripheral capillary oxygen saturation; TST: total sleep time; Percent-TST <90 NREM: percentage of total sleep time in NREM sleep with oxygen saturation below 90%; Percent TST < 90 REM: percentage of total sleep time in REM sleep with oxygen saturation below 90%; Lowest SPO2: lowest recorded oxygen saturation during sleep; REM latency: time from sleep onset to the first REM period; Arousal index: number of arousals per hour of sleep (specific to REM or NREM stages) * shows a significant p-value; p<0.05 was considered significant

Parameter	REM-OS Mean ± SD	NREM-OSA Mean ± SD	Independent t-test value	p-value
Wake after sleep onset (WASO) (mins)	119.06 ± 100.03	79.53 ± 60.29	1.854	0.149
REM-latency (mins)	126.30 ± 113.62	152.01 ± 198.28	-0.616	0.762
REM-AHI (events/hr)	24.29 ± 13.68	38.84 ± 24.23	-2.86	0.085
NREM-AHI (events/hr)	5.27 ± 2.63	35.56 ± 29.17	-5.66	0.0001*
REM-AHI/NREM-AHI (ratio)	4.89 ± 2.89	1.76 ± 2.13	4.77	0.0001*
Overall AHI (events/hr)	7.65 ± 3.09	38.61± 28.77	-5.86	0.0001*
Arousal index - NREM (events/hr)	8.53 ± 13.20	15.41 ± 15.25	-1.86	0.156
Arousal index- REM (events/hr)	9.55 ± 13.55	15.72 ± 16.36	-1.59	0.225
Mean SPO2 -NREM (%)	91.00 ± 3.31	91.70 ± 3.28	-0.82	0.425
Mean SPO2- REM (%)	86.80 ± 6.76	88.80 ± 6.77	-1.14	0.242
Percent-TST <90 NREM	67.86 ± 93.40	48.87 ± 66.77	0.90	0.536
Percent TST < 90 REM	24.11 ± 27.11	12.01 ± 13.31	2.19	0.083
Lowest SPO2 (%)	75.80± 11.09	72.05 ± 14.63	1.119	0.453

As shown in Table 5, the Mini-Cog score was significantly lower in REM OSA (1.30 ± 1.34) compared to NREM OSA (4.00 ± 1.28) (p = 0.0001). The PHQ-9 score was considerably higher in REM OSA (12.40 ± 4.20) than in NREM OSA (3.48 ± 2.70) (p = 0.0001).

**Table 5:** Mini-Cog and PHQ-9 questionnaire in REM-OSA vs NREM-OSA *shows a significant p-value REM: rapid eye movement; NREM: non-rapid eye movement; OSA: obstructive sleep apnea

Parameter	REM	NREM	t-test value	p-value
Mini-cog score	1.30 ± 1.34	4.00 ± 1.28	-7.66	0.0001*
PHQ-9 score	12.40 ± 4.20	3.48 ± 2.70	9.71	0.0001*

## Discussion

The present study compared neurocognitive function in patients with REM-predominant OSA (REM-OSA) and NREM-predominant OSA (NREM-OSA), focusing on cognition and mental status. Significant differences emerged between the two groups, particularly in neurocognitive parameters, aligning with and expanding upon existing literature [20-22], especially within South Asian populations [22].

A total of 50 patients were included, comprising 60% male patients and 40% female patients. Among patients with NREM-predominant OSA, 27 (67.5%) were male patients and 13 (32.5%) were female patients. Conversely, three (30%) were male patients among patients with REM-predominant OSA, and 7 (70%) were female patients. This gender distribution is consistent with previous reports showing that women with OSA are more likely to present with REM-predominant disease, potentially due to hormonal influences and anatomical differences in upper airway structure [20]. The study also noted gender-specific symptom patterns, with females more frequently presenting with fatigue and males with snoring. This observation supports findings from Asian studies, where cultural and biological factors may contribute to the underdiagnosis of OSA in women, as fatigue is less likely to prompt medical evaluation than snoring [21,22].

No significant age differences were found between REM-OSA and NREM-OSA groups. However, the mean BMI in REM-OSA patients was notably higher (32.5 ± 6.4 kg/m²). Prior research suggests that South Asian populations may develop OSA at lower BMI thresholds than Western cohorts [23]. A slightly higher mean heart rate was observed in REM-OSA, though not statistically significant, potentially reflecting autonomic dysregulation previously associated with this phenotype [24].

Analysis of sleep architecture revealed distinct patterns. NREM-OSA patients had a significantly higher NREM-AHI (35.56 ± 29.17 vs. 5.27 ± 2.63, p=0.0001), whereas the REM-AHI/NREM-AHI ratio was markedly higher in REM-OSA (4.89 ± 2.89 vs. 1.76 ± 2.13, p=0.0001). Despite a lower overall AHI in REM-OSA (7.65 ± 3.09 vs. 38.61 ± 28.77, p=0.0001), these patients exhibited greater neurocognitive impairment. This paradox aligns with evidence that intermittent hypoxia during REM sleep particularly affects brain areas responsible for memory and mood regulation [25].

Arousal indices and mean oxygen saturation were comparable between groups. However, the total sleep time spent with oxygen saturation below 90% (%TST <90) was higher in REM-OSA, indicating more prolonged nocturnal hypoxemia. This supports earlier findings that REM-related hypoxemia is more predictive of cognitive impairment than NREM-related events [26,27].

In our study, marked differences in neurocognitive performance were observed, particularly in functions related to memory and mood. REM-OSA patients had significantly lower Mini-Cog scores (1.30 ± 1.34 vs. 4.00 ± 1.28, p=0.0001) and higher PHQ-9 scores (12.40 ± 4.20 vs. 3.48 ± 2.70, p=0.0001), indicating more severe cognitive decline and depressive symptoms. These results align with studies linking REM-OSA to hippocampal atrophy and prefrontal cortex dysfunction-changes that impair memory and executive functioning [13,25]. The elevated PHQ-9 scores align with the well-documented bidirectional relationship between REM sleep disruption and mood disorders [28].

Globally, REM-OSA is increasingly recognized as a distinct OSA phenotype with specific neurocognitive risks [29]. In South Asian populations, delayed diagnosis due to atypical symptom presentation (e.g., fatigue) and limited access to polysomnography pose additional challenges [30]. Therefore, our study highlights the importance of refining diagnostic criteria and implementing earlier interventions for younger adults with REM-predominant OSA.

Limitations

The relatively small sample size and potential inaccuracies in BMI measurement limit the generalizability of the findings. As this was a single-center study conducted in an Indian tertiary care hospital, the results may not directly apply to populations with different demographic or cultural characteristics. The cross-sectional design precludes causal inferences and allows only the demonstration of associations between REM-predominant OSA and cognitive impairment. The absence of long-term follow-up further limits insights into the progression of neurocognitive decline and the impact of treatment interventions over time. Although validated tools such as the Mini-Cog and PHQ-9 were employed, using more comprehensive neuropsychological batteries and objective neuroimaging techniques could provide a deeper understanding of the underlying mechanisms. Potential confounding factors, including sex distribution, socioeconomic status, education level, comorbidities, and the bidirectional role of depression, may have influenced the results despite careful exclusion criteria. Additionally, reliance on a single-night polysomnography may not fully account for night-to-night variability in sleep architecture. Future studies should therefore recruit larger, multicenter cohorts with more diverse populations, adopt longitudinal designs, and incorporate advanced assessments such as multimodal neuroimaging to elucidate better the mechanisms linking REM-OSA with cognitive decline.

## Conclusions

In this cross-sectional study, REM-predominant OSA was associated with more severe neurocognitive impairment than NREM-predominant OSA, despite the latter group having higher overall AHI values. This paradox highlights the need to explore additional pathophysiological contributors beyond AHI, such as reduced intracranial oxygen supply, fragmented sleep, oxidative stress, and systemic inflammation. Given the study design, these results demonstrate association rather than causation. While adherence to CPAP therapy during early morning hours when REM sleep is more prevalent remains clinically significant, the findings should primarily be considered hypothesis-generating. Routine neurocognitive screening for all REM-predominant OSA patients cannot yet be universally recommended based on this study alone; instead, the results support the need for further large-scale, prospective studies to confirm causality and define clinical protocols.
